# Selective Axonal Expression of the Kv1 Channel Complex in Pre-myelinated GABAergic Hippocampal Neurons

**DOI:** 10.3389/fncel.2019.00222

**Published:** 2019-05-16

**Authors:** Giulia Bonetto, Bruno Hivert, Laurence Goutebroze, Domna Karagogeos, Valérie Crépel, Catherine Faivre-Sarrailh

**Affiliations:** ^1^INSERM UMR1249, Institut de Neurobiologie de la Méditerranée, Aix-Marseille Université, Marseille, France; ^2^INSERM UMR-S 1270, Institut du Fer à Moulin, Faculté des Sciences et Ingénierie, Sorbonne Université, Paris, France; ^3^Department of Basic Sciences, Institute of Molecular Biology and Biotechnology, Foundation for Research and Technology, University of Crete Medical School – University of Crete, Heraklion, Greece

**Keywords:** Caspr2, TAG-1, protein 4.1B, juxtaparanodes, parvalbumin, somatostatin, interneuron

## Abstract

In myelinated fibers, the voltage-gated sodium channels Nav1 are concentrated at the nodal gap to ensure the saltatory propagation of action potentials. The voltage-gated potassium channels Kv1 are segregated at the juxtaparanodes under the compact myelin sheath and may stabilize axonal conduction. It has been recently reported that hippocampal GABAergic neurons display high density of Nav1 channels remarkably in clusters along the axon before myelination (Freeman et al., 2015). In inhibitory neurons, the Nav1 channels are trapped by the ankyrinG scaffold at the axon initial segment (AIS) as observed in pyramidal and granule neurons, but are also forming “pre-nodes,” which may accelerate conduction velocity in pre-myelinated axons. However, the distribution of the Kv1 channels along the pre-myelinated inhibitory axons is still unknown. In the present study, we show that two subtypes of hippocampal GABAergic neurons, namely the somatostatin and parvalbumin positive cells, display a selective high expression of Kv1 channels at the AIS and all along the unmyelinated axons. These inhibitory axons are also highly enriched in molecules belonging to the juxtaparanodal Kv1 complex, including the cell adhesion molecules (CAMs) TAG-1, Caspr2, and ADAM22 and the scaffolding protein 4.1B. Here, taking advantage of hippocampal cultures from 4.1B and TAG-1 knock-out mice, we observed that 4.1B is required for the proper positioning of Caspr2 and TAG-1 along the distal axon, and that TAG-1 deficiency induces alterations in the axonal distribution of Caspr2. However, the axonal expression of Kv1 channels and clustering of ankyrinG were not modified. In conclusion, this study allowed the analysis of the hierarchy between channels, CAMs and scaffolding proteins for their expression along hippocampal inhibitory axons before myelination. The early steps of channel compartmentalization preceding myelination may be crucial for stabilizing nerve impulses switching from a continuous to saltatory conduction during network development.

## Introduction

In myelinated fibers, ion channels are targeted to precise sub-cellular compartments at the axon initial segment (AIS) and nodes of Ranvier contributing to safe action potential propagation. At the node of Ranvier, the voltage-gated Na^+^ (Nav) channels are enriched at the nodal gap to ensure saltatory conduction, while the K^+^ channels are localized at nodes and juxtaparanodes to secure spike propagation (Rasband, 1998; Devaux and Gow, 2008). On both sides of the node, the paranodal junctions restrict the lateral diffusion of Na^+^ and K^+^ channels and preclude current leakage across the paranodes (Salzer, 2008). The segregation of ion channels at the nodes of Ranvier is induced by contacts with the myelinating glial cells (Ching et al., 1999). Moreover, the localization of the Na^+^ and K^+^ channels is strongly dependent on cell adhesion molecules (CAMs) at nodes, paranodes, and juxtaparanodal regions of myelinated axons (Eshed-Eisenbach and Peles, 2013). Specifically, the nodal concentration of Na^+^ channels depends on the interactions between the axonal CAM neurofascin186 and extracellular matrix proteins (Sherman et al., 2005; Feinberg et al., 2010). Neurofascin186 clustering recruits the scaffolding proteins ankyrinG and ßIV spectrin which in turn mediate the sequestration of Nav channels. In addition, the lateral diffusion of nodal Na^+^ channels is restricted by the axo-glial junctions at paranodes, which are formed by the axonal proteins Caspr/contactin and the glial neurofascin155 (Bhat et al., 2001; Charles et al., 2002).

The trapping of voltage-gated K^+^ (Kv) Kv1.1/Kv1.2 channels at the juxtaparanodal regions depends on axo–glial interactions mediated by CAMs including TAG-1/contactin2/CNTN2 and Caspr2/CNTNAP2 (Poliak et al., 2001, 2003; Traka et al., 2003; Horresh et al., 2008). Within the axonal membrane, TAG-1 forms a *cis*-complex with Caspr2, which allows the arrangement of a ternary complex with the glial-secreted form of TAG-1 (Tzimourakas et al., 2007; Savvaki et al., 2010). Disruption of either Caspr2 or TAG-1 in knock-out (KO) mice prevents the proper clustering of Kv1 channels at juxtaparanodes (Poliak et al., 2003; Traka et al., 2003). Moreover, TAG-1-deficient animals show alteration of myelinated axon conduction in the corpus callosum (Zoupi et al., 2018), as well as behavioral deficits and defects in gating and motor coordination (Savvaki et al., 2008). Similarly, the loss of Caspr2 is associated with defects in the propagation of action potentials along myelinated axons in the corpus callosum, with a slow-down of the repolarisation phase (Scott et al., 2017).

A number of scaffolding proteins, including band 4.1B, αII, and ßII spectrin are expressed at paranodes and juxtaparanodes (Denisenko-Nehrbass et al., 2003; Ogawa et al., 2006; Zhang et al., 2013). In 4.1B-null mice the accumulation of TAG-1, Caspr2, and Kv1 at juxtaparanodes is altered, indicating the crucial role of this protein in the formation of the juxtaparanodal domain (Horresh et al., 2010; Buttermore et al., 2011; Cifuentes-Diaz et al., 2011; Einheber et al., 2013). A dynamic and precise sub-compartmentalization of Kv1 channels that may help to regulate the conduction occurs in developing myelinated axons (Vabnick et al., 1999). Precisely, Kv1 channels and Caspr2 are first enriched at paranodes and progressively restricted to juxtaparanodes, while they could be seen transiently trapped between heminodes and at the newly formed nodal zone. In contrast, the distribution of the scaffolding protein 4.1B is restricted to internodes and preferentially co-localized with Caspr at paranodes (Hivert et al., 2016). The transient localization of Kv1 channels at nodes and paranodes may be directly involved in speeding repolarisation to allow trains of action potentials (Vabnick et al., 1999).

In inhibitory neurons, the myelination processes as well as the mechanisms leading to the segregation of ion channels at the nodes of Ranvier have been poorly investigated. It has been recently reported that pre-myelinated hippocampal GABAergic neurons, including parvalbumin (PV) and somatostatin (SST) cells, display high density of Nav1 channels associated with ankyrinG in clusters (the so-called “pre-nodes”) along the axon (Freeman et al., 2015). Adhesive contact with ensheathing myelinating cells is a prerequisite for the nodal trapping of Na^+^ channels in pyramidal neurons whereas pre-nodal clusters can be selectively induced in GABAergic neurons by oligodendroglial secreted factors. Interestingly, the presence of Nav1 clusters is correlated with an acceleration of conduction in pre-myelinated inhibitory axons (Freeman et al., 2015).

In the present study, we examine the distribution of the Kv1 complex in hippocampal pre-myelinated inhibitory neurons. We show that GABAergic neurons, namely the PV and SST cells, selectively display high concentration of Kv1 channels all along their axon and the associated molecules, TAG-1, Caspr2, ADAM22, and protein 4.1B. Furthermore, we demonstrate that in these pre-myelinated inhibitory neurons, TAG-1 is required for the proper distribution of Caspr2, while 4.1B is necessary for the correct localization of both Caspr2 and TAG-1. Interestingly, we found that TAG-1 expression *in vivo* is constrained to specific CA1 hippocampal layers; it is selective to the SST cells in the stratum oriens and the PV cells in the stratum pyramidale and may be possibly related to a pre-myelinated phenotype. The specific expression of CAMs associated with the Kv1 channels in the GABAergic neurons may help to secure conduction during network development.

## Materials and Methods

### Animals

The care and use of rats and mice in all experiments were carried out according to the European and Institutional guidelines for the care and use of laboratory animals and approved by the local authority (laboratory’s agreement number D13-055-8, Préfecture des Bouches du Rhône). The following rat and mouse strains were used in this study: Wistar rats and C57bl/6 mice (Janvier Breeding Center), *Tag-1* KO mice (Traka et al., 2003), and 4.1B KO mice (Cifuentes-Diaz et al., 2011).

### Cell Culture

Primary mixed hippocampal cell cultures were prepared from embryonic day 18 Wistar rats. Hippocampi were collected in Hanks’ balanced salt solution, dissociated with trypsin and plated at a density of 1.2 10^5^ cells/cm^2^ on poly-L-lysine (Sigma-Aldrich, Merck) coated coverslips. Hippocampal neurons were cultured in Neurobasal supplemented with 2% B-27, 1% penicillin-streptomycin and 0.3% glutamine in a humidified atmosphere containing 5% CO2 at 37°C. The mixed hippocampal cultures were maintained for 3–4 weeks *in vitro* and contained astrocytes and oligodendrocytes. Unless specified, all culture reagents were purchased from Gibco, Thermo Fisher Scientific. Once a week half of the culture medium was replenished. Hippocampal cell cultures were prepared from embryonic day 16 wild type, *Tag-1* KO (Traka et al., 2003) or 4.1B KO mice (Cifuentes-Diaz et al., 2011) using the same protocol. At least, three different hippocampal cell cultures were performed from wild type and KO mice and processed in parallel on the same days for immunofluorescence staining.

### Antibodies and Immunofluorescence Staining

The following primary antibodies were used: rabbit antiserum against protein 4.1B (Cifuentes-Diaz et al., 2011), rabbit anti-TAG-1 TG3 (Buttiglione et al., 1998), and rabbit anti-ankyrinG, a gift from Dr. Gisèle Alcaraz. Human anti-Caspr2 antiserum was previously described (Pinatel et al., 2015). Mouse anti-panNav mAb (clone K58/35) was purchased from Sigma, chicken anti-MAP2 antibody (ab5392) and rat anti-MBP mAb (ab7349) from Abcam, rabbit anti-Prox1 antibody (ab5475) from Millipore, goat anti-PV antibody (PVG-214) from Swant, goat anti-SST mAb (sc55565) from Santa-Cruz. Mouse anti-Kv1.1 (clone K20/78), anti-Kv1.2 (clone K14/16), anti-Kv1.4 (clone K13/31), anti-ADAM22 (clone N46/30), and anti-ankyrinG (clone N106/36) mAbs were obtained from NeuroMab (UC Davis/NIH NeuroMab Facility). Mouse anti-TAG-1 (1C12) and anti-GAD65 (GAD6) mAbs were from Developmental Studies Hybridoma Bank. AlexaFluor-405, -488, -568 and -647-conjugated secondary antibodies were purchased from Molecular Probes.

Live immunostaining was performed using mouse anti-TAG-1 1C12 (1:2000), rabbit anti-TAG-1 TG3 (1:400), or human anti-Caspr2 (1:400) antibodies for 30 min and with secondary antibodies (1:800) for 30 min diluted in culture medium at room temperature. Cells were fixed with 1 or 4% paraformaldehyde in PBS for 10 min and permeabilized with 0.1% Triton-X100 for 10 min. Immunofluorescence staining on fixed neurons was performed using rabbit anti-ankyrinG (1:400), rabbit anti-4.1B (1:2000), rabbit anti-prox1 (1:2000), chicken anti-MAP2 (1:10,000) antibodies, rat anti-MBP mAb (1:200), mouse anti-panNav (1:500), anti-Kv1.1, anti-Kv1.2, anti-Kv1.4, anti-ADAM22, anti-ankyrinG (1:100) mAbs for 60 min and with AlexaFluor-conjugated secondary antibodies for 30 min diluted in PBS with 3% bovin serum albumin. Immunostaining with goat anti-PV (1:500) or mouse anti-SST (1:200) antibodies was performed overnight at 4°C.

### *In vivo* Study and Immunohistochemistry

For the *in vivo* study, P21 (*n* = 3) rats were deeply anesthetized with a mix of ketamine–xylazine (Vetoquinol) and then transcardially perfused with PBS followed by 4% paraformaldehyde in PBS. Brains were removed and placed in the same fixative for 30 min, then cryoprotected by infiltration in 30% sucrose overnight, embedded in 7.5% gelatin-15% sucrose, and immediately frozen in a dry ice-isopentane bath. Thirty micron-thick cryostat sections were mounted on SuperfrostVR Plus microscope slides (Thermo Fisher Scientific), permeabilized by immersion in ice-cold acetone at -20°C for 10 min, blocked for 1 h in 5% bovine serum albumin containing 0.5% Triton X-100 in PBS, and incubated overnight at 4°C with combinations of the following primary antibodies: goat anti-PV (1:500), goat anti-SST (1:500), mouse anti-TAG-1 (1C12, 1:2000), human anti-Caspr2 (1:200), mouse anti-Kv1.2 (1:100), rabbit anti-ankyrinG (1:400), rat anti-MBP (1:200). Slides were then washed and incubated with the appropriate AlexaFluor-conjugated secondary antibodies (1:800) for 2 h. Slides were covered with Vectashield mounting medium (Vector Laboratory), which contains DAPI to visualize cell nuclei.

### Image Acquisition and Statistical Analysis

Image acquisition was performed on a Zeiss laser-scanning microscope LSM780 equipped with 63× 1.32 NA oil-immersion objective for cell culture and 20× or 63× objectives for hippocampal slice imaging. Images of AlexaFluor-stained cells were obtained using the 488 nm band of an Argon laser and the 405, 568, and 647 nm bands of a solid-state laser for excitation. Fluorescence images were collected automatically with an average of two-frame scans at airy 1. Maximum intensity projection of images and plot profiles of immunofluorescence intensity (10 pixels width) were carried out using ImageJ software (NIH). Images are single confocal sections unless the number of z-steps is indicated.

The percentage of SST and PV neurons positive for TAG-1, Caspr2, and Kv1.2 was determined by examining at least 50 neurons on 2 coverslips per condition (wild type, TAG-1 KO and 4.1B KO mice). 4-z step confocal sections (410 nm) were acquired with the same settings (laser intensity and gain) and maximum intensity projections were generated before analysis. Results were expressed as mean ± SEM of at least three independent experiments. Statistical analyses were performed using the GraphPad Prism software. The data normal distribution was tested using d’Agostino and Pearson’s test. The Student’s paired *t*-test or the one-way analysis of variance (ANOVA), followed by Dunnett *post hoc* test was performed.

## Results

### The Kv1.2 Subunits Are Highly Expressed Along Inhibitory Axons in Hippocampal Cultures

**FIGURE 1:**
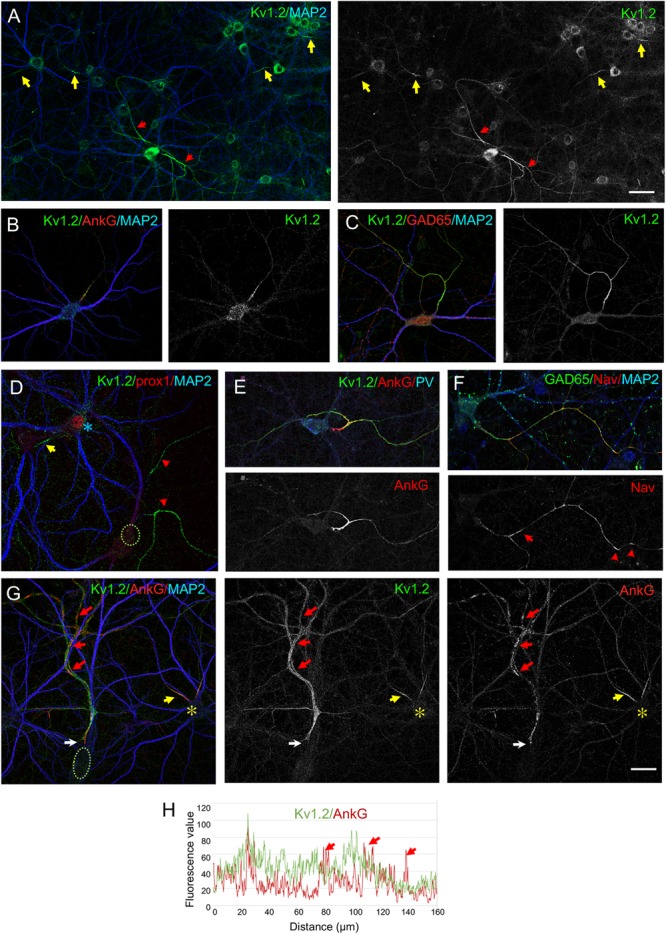
Cell-type specific distribution of Kv1.2 in cultured hippocampal neurons. Hippocampal neurons at DIV21 were fixed, permeabilized and immunostained for Kv1.2 (green). MAP2 (blue) is used as a somato-dendritic marker and ankyrinG (AnkG) (**B,E,G**, red) as a marker of AIS. Kv1.2 is restricted at the AIS of most neurons (yellow arrows in panel **A,B**) or distributed all along the axon in a subpopulation of neurons (red arrows in panel **A**) identified as GABAergic neurons using GAD65 labeling (**C**, red). **(D)** Granule cells positive for prox1 (blue asterisk) display Kv1.2 localized at the AIS. **(E,F)** GABAergic neurons including PV^+^ neurons (**E**, blue) exhibit high expression of Kv1.2 all along their axon and clusters of Na^+^ channels labeled using anti-panNav mAb (**F**, red). **(G,H)** Fluorescence intensity profiles were generated starting from the AIS (**G**, white arrows) following the axon. The axonal clusters of ankyrinG are indicated with red arrows **(G,H)**. Kv1.2 is co-localized with ankyrinG at the AIS, but do not co-cluster with ankyrinG along the axon. Note that Kv1.2 is restricted to the AIS (**G**, yellow arrow) in a neighboring neuron (asterisk). Scale bar: 50 μm in panel **(A)**; 20 μm in panel **(B–G)**.

We examined the distribution of the Kv1 channels in hippocampal cell culture at Day *in vitro* DIV21, a representative day before myelination onset. It has been reported that Kv1.2 is concentrated at the AIS in subpopulations of hippocampal neurons after DIV10 (Ogawa et al., 2008; Sanchez-Ponce et al., 2012). Here, we showed that Kv1.2 immunostaining was restricted at the AIS in some neurons (Figure 1A,B, yellow arrows). Kv1.2 was detected at the AIS of granule cells identified using immunostaining for the transcription factor prox1 (Figure 1D, yellow arrow). Strikingly, the Kv1.2 channels were also strongly expressed along the axon in some neurons identified as GABAergic neurons using immunostaining for GAD65 (Figure 1A,C, red arrows). We showed that these neurons were either PV^+^ or SST^+^ interneurons as illustrated for PV^+^ neurons in Figure 1E. The inhibitory axons displayed distal clusters of Nav channels and ankyrinG (Figure 1F,G, red arrows) as it has been described specifically in pre-myelinated GABAergic neurons in hippocampal cultures (Freeman et al., 2015). We analyzed more precisely the distribution of Kv1.2 relatively to the ankyrinG clusters along the inhibitory axons at DIV21. As shown in Figure 1G, immunostaining for Kv1.2 after fixation and permeabilization indicated that these channels were enriched at the AIS co-localized with ankyrinG and homogenously distributed along the axon. Plot profile analysis illustrates that Kv1.2 was concentrated at the AIS together with ankyrinG and uniformly present along the axon irrespectively to peaks corresponding to ankyrinG clusters (Figure 1H, red arrows).

We noticed that the GABAergic neurons showing Kv1.2 distribution all along their axon also exhibited high expression of the Kv1.2 channel at the level of their soma by comparison with excitatory neurons that displayed AIS-restricted expression of Kv1.2 (Figure 1A). We estimated the immunofluorescence intensity of Kv1.2 in the soma of GAD65-positive neurons (*n* = 30) relatively to those of other neurons in the same area (*n* = 93) and observed that it was increased by 33 ± 4%. These data suggest that the axonal distribution of Kv1.2 channels may be correlated with the level of expression in the cell body.

**FIGURE 2:**
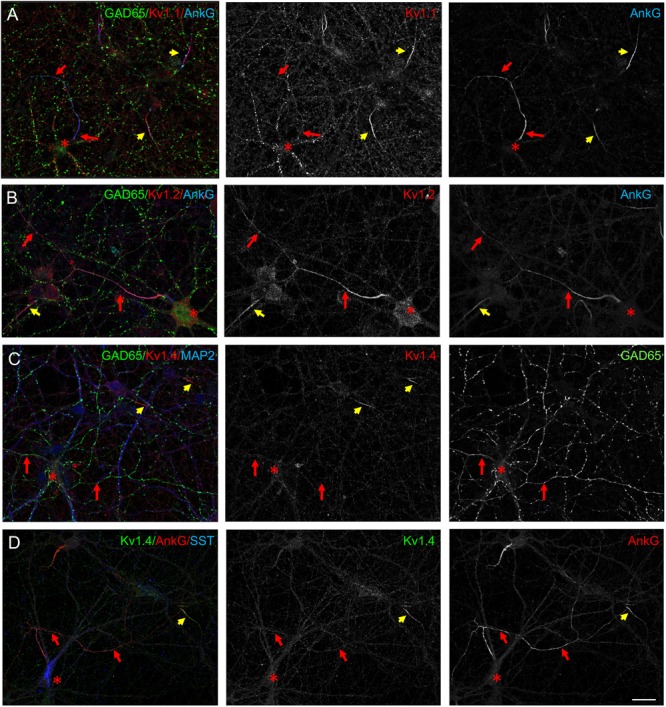
Expression of Kv1 subunits in cultured hippocampal neurons. Cultured hippocampal neurons at DIV21 were immunostained for Kv1.1 **(A)**, Kv1.2 **(B)**, or Kv1.4 **(C,D)**. Double-staining for GAD65 (**A–C**, green) or SST (**D**, blue) and ankyrinG (**A–C**, blue; **D**, red). Kv1.1, Kv1.2, and Kv1.4 are expressed at the AIS of excitatory neurons co-localized with ankyrinG (yellow arrows) and negative for SST or GAD65. **(A)** Kv1.1 is faintly detected along the axon (red arrows) and is present in intracellular vesicles in inhibitory neurons. **(C,D)** Kv1.4 is not detected along the inhibitory axons (red arrows). The cell bodies of SST- and GAD65-positive neurons are indicated with red asterisks. Scale bar: 20 μm.

Kv1 channels exist as homomeric and heteromeric complexes in neurons and distinct Kv1 channels could be selectively addressed at the axonal membrane based on their subunit composition (Jenkins et al., 2011). In addition to Kv1.2, the Kv1.1, and Kv1.4 subunits have been reported to be concentrated at the AIS of cultured hippocampal neurons (Ogawa et al., 2008). Indeed, we observed that Kv1.1 was expressed at the AIS of excitatory neurons (Figure 2A, yellow arrows). However, Kv1.1 was weakly expressed at the AIS and along the axons of GAD65-positive inhibitory neurons by comparison with Kv1.2 (Figure 2A,B) whereas it was mainly detected as vesicles in the somato-dendritic compartment of these cells (Figure 2A). The Kv1.4 subunit was colocalized with ankyrinG at the AIS of excitatory neurons (Figure 2C,D, yellow arrows), but very faintly expressed in GAD65- or SST-positive neurons (Figure 2C,D, red arrows). Altogether, these results suggest that Kv1 channels expressed in inhibitory axons in hippocampal cell cultures are composed mainly of Kv1.2 subunits.

**FIGURE 3:**
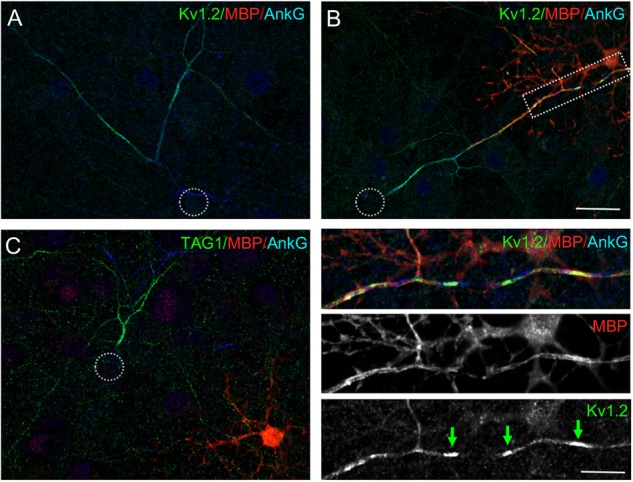
Kv1.2 and TAG-1 are highly expressed along axons before myelination. Cultured hippocampal neurons at DIV21 were immunostained for Kv1.2 (**A,B**, green) or TAG-1 (**C**, green), MBP (red) as a marker of oligodendrocytes and ankyrinG (blue). **(A,C)** A number of unmyelinated neurons are labeled for Kv1.2 or TAG-1 all along their axons. **(B)** Few neurons are myelinated and show high density of Kv1.2 close to ankyrinG clusters under myelinated segments (green arrows in the inset). Scale bar: 20 μm; 10 inset μm.

Next, we asked whether the high level of Kv1.2 along inhibitory axons may be correlated with myelination or occurred before myelination onset using immunostaining for MBP as a marker of myelinating oligodendrocytes (Figure 3A). Mixed hippocampal cultures were performed in standard conditions but with plating at high density. These cultures contained glial cells, including few oligodendrocytes identified using immunostaining for the myelin basic protein (MBP) (Figure 3, red). However, at DIV 21, only very few neurons presenting high level of Kv1.2 along their axon were myelinated and in this case Kv1.2 only formed clusters along the myelinated segments (Figure 3B, green arrows in inset).

### TAG-1, Caspr2, ADAM22, and Protein 4.1B Are Selectively Expressed Along Inhibitory Axons in Cultured Hippocampal Neurons

**FIGURE 4:**
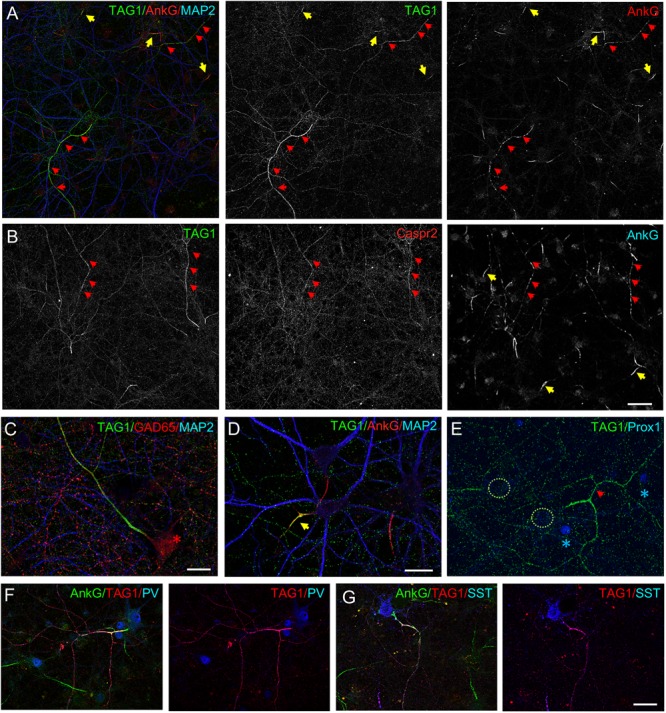
Cell-type specific expression and distribution of TAG-1 and Caspr2 in cultured hippocampal neurons. Hippocampal neurons at DIV21 were surface labeled using anti-TAG-1 mAb (**A**, **C–G**) or double-labeled for TAG-1 and Caspr2 **(B)**. Cells were fixed and permeabilized before immunostaining for intracellular markers. **(A,B)** In some neurons, TAG-1 and Caspr2 are expressed all along the axons which display clusters of ankyrinG (red arrows). TAG-1 is also restricted at the AIS in a subpopulation of neurons (yellow arrows in panel **A**) as illustrated at high magnification **(D)** and is not expressed in granule cells labeled for prox1 (**E**, blue asterisks). TAG-1 is highly concentrated along the axon of GABAergic neurons (**C**, red asterisk) including PV-positive (**F**, blue) and SST-positive (**G**, blue) inhibitory neurons. Single confocal section **(A–D)** or maximum intensity of confocal images (4-z steps of 440 nm) in panel **(F,G)**. Scale bar: 50 μm in panel **(A,B)**; 20 μm in panel **(C–G)**.

We investigated whether the cell adhesion and scaffolding molecules associated with the Kv1 channels at the juxtaparanodes in myelinated fibers may be also selectively expressed by inhibitory neurons. We previously reported that Caspr2 is preferentially expressed by GABAergic neurons in hippocampal cell cultures. Using live immunostaining with anti-Caspr2 antibodies from patients affected by autoimmune encephalitis, we have shown that Caspr2 is detected all along the axolemma including at the presynaptic terminals of inhibitory neurons (Pinatel et al., 2015). Here, we investigated the cell-type specific expression of TAG-1, which interacts both in *cis* and in *trans* with Caspr2 and may be either *cis*-associated with Caspr2 along the inhibitory axons or *trans*-interacting at the post-synapse with presynaptic Caspr2 (Traka et al., 2003; Savvaki et al., 2010; Pinatel et al., 2015). Live immunostaining was performed using mouse anti-TAG-1 1C12 mAb at DIV21. As observed for Kv1.2 channels, TAG-1 was strongly expressed along the axon of some neurons, which displayed ankyrinG at the AIS as well as organized in regularly spaced clusters along the entire axonal length (Figure 4A,B, red arrowheads). Surface double-immunostaining was performed using anti-TAG-1 mAb and human anti-Caspr2 antibodies, and indicated that the two CAMs were co-expressed along the same axons (Figure 4B). We hypothesized that these axons may belong to inhibitory neurons since exhibiting axonal clusters of ankyrinG. This was confirmed using double-immunostaining for TAG-1 and GAD65 (Figure 4C). In addition, TAG-1 and Caspr2 were co-localized at the inhibitory pre-synaptic terminals surrounding the soma of pyramidal cells (Supplementary Figure S1). Next, using immunostaining with antibodies directed against SST and PV, we determined that both subtypes of inhibitory cells displayed high level of expression of TAG-1 along their axons (Figure 4F,G). As observed for Kv1.2, we noticed that the axonal distribution of TAG-1 along inhibitory axons also occurred before myelination (Figure 3C).

In addition to be highly expressed along inhibitory axons, TAG-1 was also expressed in a subpopulation of neurons at the AIS (approximately 30%) (Figure 4A,D, yellow arrows). In contrast to what has been observed for Kv1.2 (Figure 1D), the granule cells identified with the marker prox1 were negative for TAG-1 (Figure 4E).

**FIGURE 5:**
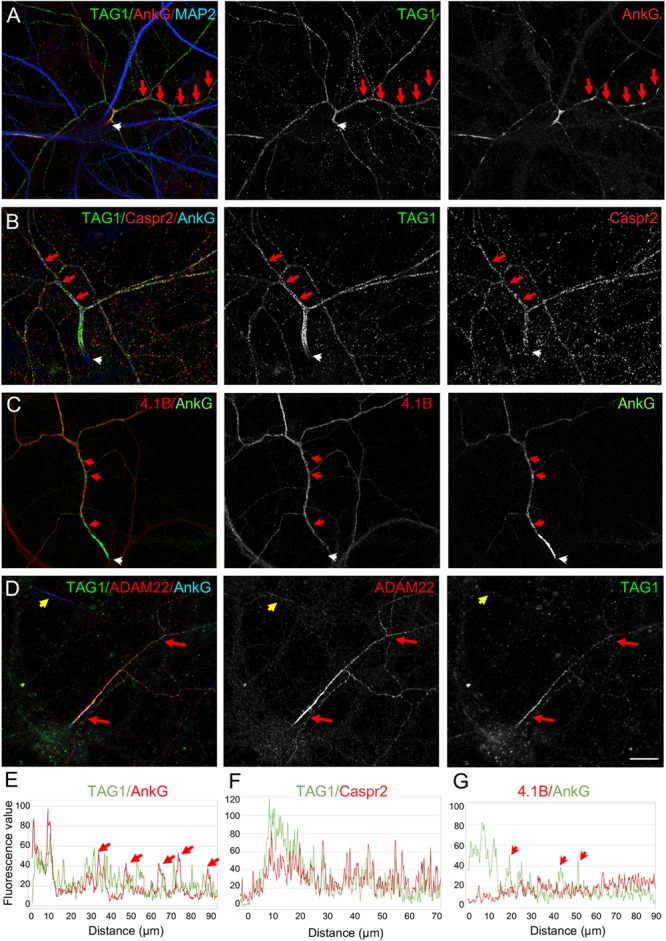
Subcellular distribution of TAG-1, Caspr2, protein 4.1B, and ADAM22 in cultured hippocampal neurons. Hippocampal neurons at DIV21 were labeled for TAG-1 **(A)**, TAG-1 and Caspr2 **(B)**, 4.1B **(C)**, or TAG-1 and ADAM22 **(D)**. Axons exhibiting clusters of ankyrinG were identified as inhibitory axons (**A–D**, red arrows). Immunolabelling for TAG-1 and Caspr2 was performed on live cells. **(A,B)** TAG-1 is enriched at the AIS whereas Caspr2 is evenly distributed along the axon. **(C)** Protein 4.1B is distributed along the axon and excluded from the AIS and ankyrinG clusters. **(D)** ADAM22 is enriched at the AIS and colocalized with TAG-1 all along the axon in neurons that display distal clusters of ankyrinG and is also detected at the AIS of other subtypes of neurons (yellow arrow). **(E–G)** Fluorescence intensity profiles were generated starting from the AIS (white arrows) following the axons in panel **(A–C)**. The axonal clusters of ankyrinG are indicated with red arrows. TAG-1 is co-localized with ankyrinG at the AIS, but do not co-cluster with ankyrinG along the axon **(E)**. Caspr2 and protein 4.1B are not enriched at the AIS **(F,G)**. Scale bar: 20 μm.

We then investigated more precisely the relative distribution of TAG-1 and Caspr2 along inhibitory axons at DIV21 and observed that the two CAMs were strongly co-localized (Figure 5B). However, only TAG-1 was enriched at the AIS as illustrated by the plot profile (Figure 5A,B,E,F). Since protein 4.1B is known to bind the cytoplasmic tail of Caspr2 and is required for the recruitment of the juxtaparanodal complex in myelinated fibers, we also analyzed its distribution and found that it was present along the distal axon and faintly expressed at the AIS of inhibitory neurons (Figure 5C,G). Similarly to TAG-1 and Caspr2, ADAM22, another CAM associated with the Kv1 complex at the AIS (Ogawa et al., 2008; Hivert et al., 2019) was found to be preferentially expressed all along inhibitory axons in mature cultured hippocampal neurons (Figure 5D). ADAM22 appeared to be enriched at the AIS of inhibitory neurons, in a manner similar to TAG-1 (Figure 5D, red arrows).

### Interdependent Expression of Caspr2, TAG-1, and Protein 4.1B in Axons of SST and PV Neurons

**FIGURE 6:**
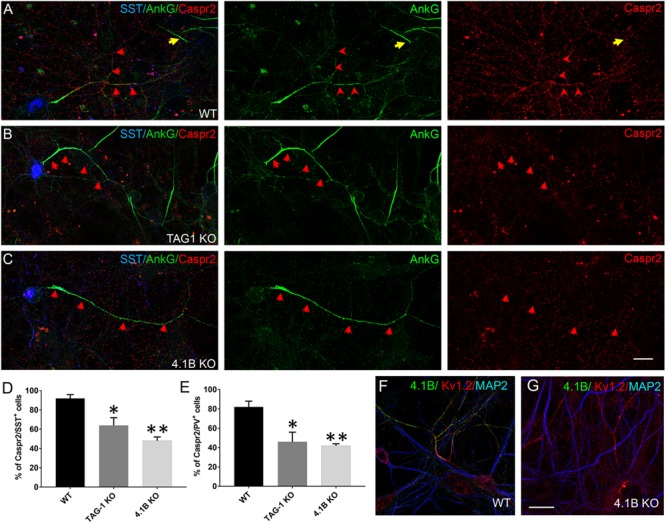
Caspr2 expression is disorganized in inhibitory neurons from TAG-1 or 4.1B KO mice. **(A–C)** Hippocampal neurons from wild type (WT) **(A)**, TAG-1 **(B)**, or 4.1B KO mice **(C)** at DIV21 were surface labeled with human anti-Caspr2 antibodies (red). Cells were then fixed, permeabilized and immunostained with mouse anti-ankyrinG (green) and goat anti-SST (blue) antibodies. Axons of SST^+^ cells are indicated with red arrows. The AIS of one SST-negative cell is indicated with a yellow arrow in panel **(A)**. Maximum intensity of confocal images (4-z steps of 410 nm). **(D,E)** Quantification of the percentage of SST^+^ **(D)** or PV^+^ **(E)** neurons that were labeled for Caspr2. The percentage of neurons showing Caspr2 axonal staining is significantly reduced in cells from either TAG-1 or 4.1B KO mice. Mean ± SEM of three independent experiments. For each experiment, at least 50 neurons were analyzed. For the statistics, one-way ANOVA with *post hoc* Dunnett test was used. Comparison with wild type group: ^∗^*p* < 0.05 and ^∗∗^*p* < 0.01. **(F,G)** Neurons from wild type **(F)** or 4.1B KO **(G)** mice immunostained using rabbit anti-4.1B (green), chicken anti-MAP2 (blue), and mouse anti-Kv1.2 (red) antibodies. Scale bar: 20 μm.

To determine whether the proteins of the Kv1 complex are interdependent for their enrichment along hippocampal inhibitory axons, we analyzed their expression taking advantage of KO mice for TAG-1 or 4.1B protein. Hippocampal cell cultures from wild type, TAG-1 KO and 4.1B KO mouse embryos were performed in parallel on the same day. As expected, we observed the absence of immunostaining for TAG-1 or 4.1B in hippocampal neurons from TAG-1 or 4.1B KO mice, respectively, confirming the specificity of immunostaining in wild type neurons (Figure 6F,G, 7C). Live immunostaining at DIV21 indicated that the cell surface expression of Caspr2 was strongly altered in the inhibitory neurons from either TAG-1 or 4.1B KO mice, as shown for SST^+^ neurons (Figure 6A–C). Specifically, 92% of SST^+^ neurons were positive for Caspr2 in the wild type, whereas only 64% or 48% of them expressed Caspr2 in the TAG-1 or 4.1B KO hippocampal cultures, respectively (Figure 6D). Similarly, only half of the PV^+^ neurons expressed Caspr2 in the TAG-1 and 4.1B KO (46 and 42%, respectively) by comparison with the wild type cultures (82%, Figure 6E). These data indicate that TAG-1 and 4.1B proteins are necessary for the proper expression of Caspr2 in inhibitory neurons, suggesting that Caspr2 may be stabilized in a preformed complex along the axonal membrane before myelination.

**FIGURE 7:**
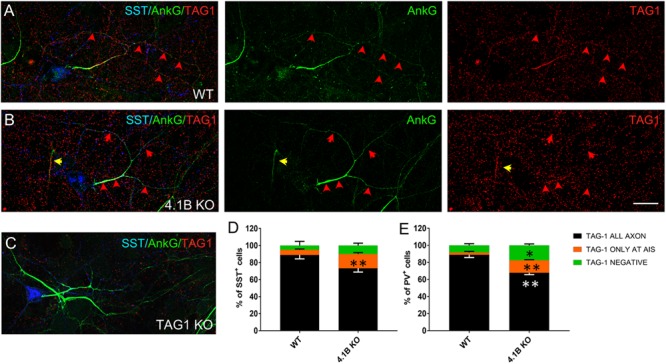
TAG-1 displays an abnormal distribution in inhibitory neurons from 4.1B KO mice. **(A–C)** Hippocampal neurons from wild type **(A)**, 4.1B KO **(B)**, or TAG-1 KO **(C)** mice were surface labeled at DIV21 for TAG-1 (red). Cells were fixed, permeabilized and immunostained with mouse anti-ankyrinG (green) and goat anti-SST (blue) antibodies. TAG-1 is enriched all along the axon in wild type inhibitory neurons **(A)**, while it is mainly expressed at the AIS in cells derived from 4.1B KO mice **(B)**. The AIS of one SST-negative cell is indicated with a yellow arrow in panel **(B)**. Note that TAG-1 immunostaining is reduced to background in neurons from TAG-1 KO mice **(C)**. Maximum intensity of confocal images (4-z steps of 410 nm). **(D,E)** Quantification of the percentage of SST^+^ **(D)** or PV^+^ **(E)** neurons showing TAG-1 all along the axon (black), only at the AIS (restricted to a distance of 50 μm from the soma, orange) or completely absent (green). Mean ± SEM of three independent experiments. For each experiment, at least 50 neurons were analyzed. For statistics, the paired *t*-test was used. Comparison with wild type group: ^∗^*p* < 0.05 and ^∗∗^*p* < 0.01. Scale bar: 20 μm.

Next, we analyzed the axonal surface expression of TAG-1 in hippocampal cultures from 4.1B KO mice (Figure 7). Remarkably, we observed that in some inhibitory neurons the protein was totally missing, whereas in other cells TAG-1 expression was restricted to the AIS as shown for SST^+^ neurons (Figure 7B, red arrows). Precisely, TAG-1 expression was detected only at the AIS in 16.7% of the SST^+^ neurons in the 4.1B KO versus 6% in wild type (Figure 7D). Likewise, TAG-1 was restricted at the AIS in 14.6% of the PV^+^ neurons in 4.1B KO versus 2.7% in the wild-type (Figure 7E). Since the axonal expression of Caspr2 is altered in the absence of protein 4.1B, altogether these data suggest that the association of TAG-1 in complex with Caspr2 and 4.1B may modulate the distal distribution of the protein along the inhibitory axons.

### Kv1.2 Distribution Is Not Altered in Knock-Out Mice for TAG-1 or Protein 4.1B

**FIGURE 8:**
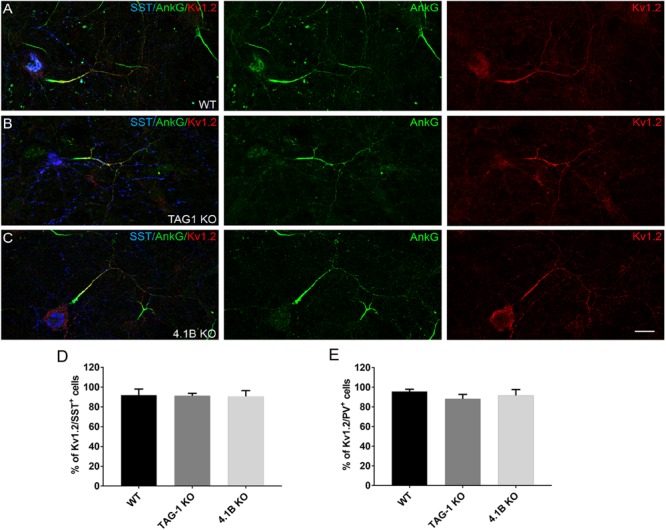
The distribution of Kv1.2 is not altered in inhibitory neurons from TAG-1 or 4.1B KO mice. **(A–C)** Hippocampal neurons at DIV21 immunostained for ankyrinG (green), Kv1.2 (red), and SST (blue). Kv1.2 is expressed all along the axons of inhibitory neurons from wild type **(A)**, TAG-1 **(B)**, or 4.1B KO mice **(C)**. Maximum intensity of confocal images (4-z steps of 410 nm). **(D–E)** Quantification of inhibitory neurons showing double-staining for SST **(D)** or PV **(E)** and Kv1.2. Mean ± SEM of three independent experiments. For each experiment, at least 50 neurons were analyzed. For the statistics, one-way ANOVA with *post hoc* Dunnett test was used and no statistical significance was found. Scale bar: 20 μm.

The distribution of Kv1 channels at the juxtaparanodes is strongly altered in myelinated axons of the KO mice for TAG-1 or 4.1B (Traka et al., 2003; Cifuentes-Diaz et al., 2011; Hivert et al., 2016). It was therefore important to analyze whether the expression or distribution of Kv1.2 could be impaired at an early step in pre-myelinated GABAergic axons deficient for TAG-1 or 4.1B. We did not observe any difference in the expression of Kv1.2 either in SST^+^ or PV^+^ neurons deficient for TAG-1 or protein 4.1B, compared to the wild type situation as illustrated for SST^+^ neurons (Figure 8A–C). Kv1.2 was expressed in approximately 90% of the SST^+^ and PV^+^ neurons in the cultures from wild type, TAG-1 or 4.1B KO mice at DIV21 (Figure 8D,E). Furthermore, the high expression of Kv1.2 channels was correlated with the presence of ankyrinG clusters both in the wild type and mutant mice (Figure 8A–C). All together, these results point toward a mechanism for recruiting Kv1 channels along pre-myelinated inhibitory axons, which is independent from TAG-1 and protein 4.1B.

### *In vivo* Expression of the Cell Adhesion Molecules Associated With the Kv1 Channels in the Developing Hippocampus

**FIGURE 9:**
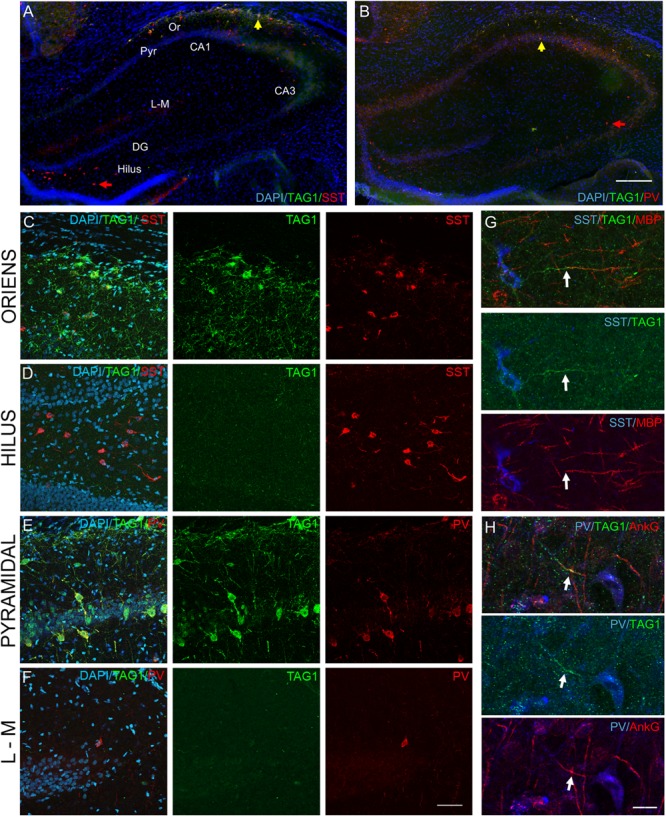
TAG-1 is expressed in subtypes of PV^+^ and SST^+^ inhibitory neurons in the hippocampus. **(A,B)** Coronal hippocampal cryosections from postnatal day 21 (P21) rat at low magnification, stained for the nuclear marker DAPI, TAG-1 (green) and SST **(A)** or PV **(B)**. The indicated hippocampal areas, i.e., the stratum oriens (Or), the pyramidal layer (Pyr), the stratum lacunosum-moleculare (L-M) and the hilus, are shown at high magnification in panel **(C–F)**. **(C–F)** Coronal hippocampal cryosections from P21 rat labeled for TAG-1 (green), SST (red, **C,D**) or PV (red, **E,F**), and DAPI. TAG-1 is selectively expressed by SST^+^ and PV^+^ inhibitory neurons in specific layers of the CA1 hippocampus, the stratum oriens for SST^+^ neurons (**C** and yellow arrow in panel **A**) and the pyramidal layer for PV^+^ cells (**E** and yellow arrow in panel **B**). TAG-1 is not expressed in other areas of the hippocampus, such as in the SST^+^ cells in the hilus of the dentate gyrus (**D** and red arrow in panel **A**) or the PV^+^ cells in the stratum lacunosum-moleculare **(F)** or CA3 pyramidal layer (red arrow in panel **B**). **(G,H)** Sagittal hippocampal cryosections from P21 rat labeled for SST, TAG-1, and MBP as a marker of myelin **(G)** or PV, TAG-1, and ankyrinG **(H)**. TAG-1 is localized at the AIS (white arrows) in both SST^+^ and PV^+^ inhibitory neurons in the CA1 hippocampal region. Note that the SST^+^ neuron positive for TAG-1 is myelinated. Wide-field **(A,B)** and confocal **(C–H)** microscopy images. Maximum intensity of 6-z steps of 830 nm **(C–F)** or 3-z steps of 1.38 μm **(G)** or 1 μm **(H)**. Scale bar: 80 μm in panel **(A,B)**; 50 μm in panel **(C–F)**; 15 μm in panel **(G,H)**.

To evaluate the physiological relevance of our observations *in vitro*, we further addressed whether GABAergic neurons may also selectively express the Kv1 complex proteins in the developing hippocampus. The onset of myelination of GABAergic neurons in the hippocampus was reported from P14 (Freeman et al., 2015). Using immunostaining on hippocampal tissue sections from rats at post-natal day 21 (P21), we analyzed the expression of TAG-1 in SST^+^ and the PV^+^ inhibitory neurons (Figure 9). We observed that the SST^+^ neurons located in the stratum oriens of the CA1 region were positive for TAG-1 (42.1 ± 4.3%, *n* = 3) (Figure 9A,C). In contrast, SST^+^ cells in other hippocampal regions, such as the hilus of the dentate gyrus, were negative for TAG-1 (Figure 9A,D). The PV^+^ neurons expressed TAG-1 within the pyramidal layer (69.9 ± 8.3%, *n* = 3) (Figure 9B,E) while they were negative in other areas, such as the stratum lacunosum moleculare (Figure 9F). In addition, these subtypes of inhibitory neurons were negative for TAG-1 within the other hippocampal areas i.e., the CA3 and the dentate gyrus (Figure 9A,B). We showed that TAG-1 was enriched at the AIS of SST^+^ cells proximal to the myelin segment immunostained for MBP (Figure 9G) and also localized at the AIS of PV^+^ cells using double-staining for ankyrinG (Figure 9H).

**FIGURE 10:**
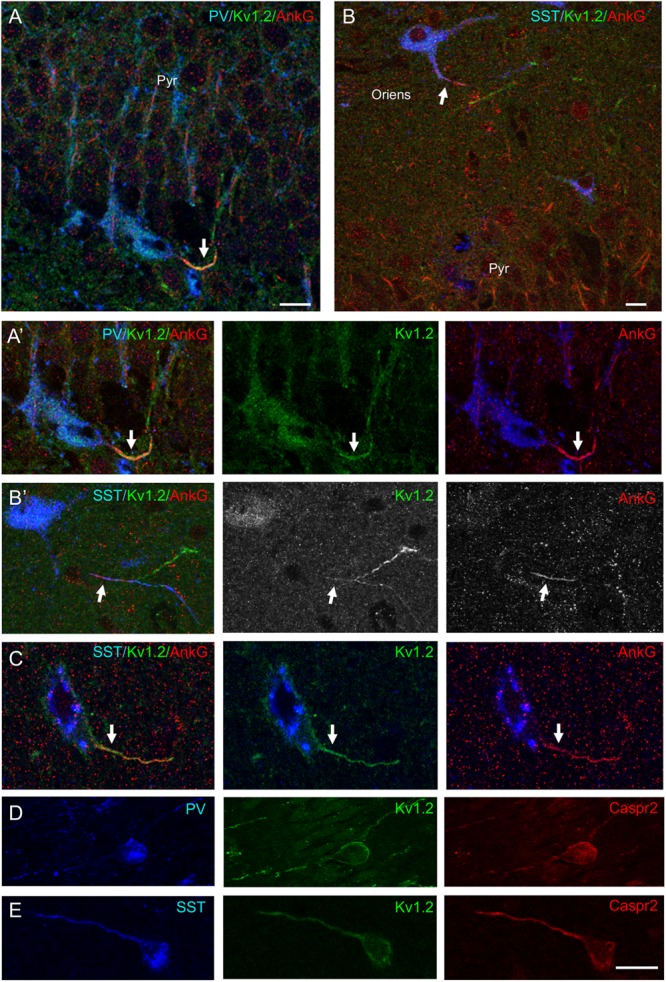
Kv1.2 is expressed at the AIS of PV^+^ and SST^+^ inhibitory neurons in the CA1 layer of the rat hippocampus. Sagittal hippocampal cryosections from P21 rat labeled for PV (**A**, blue) or SST (**B,C**, blue), ankyrinG (red) or Caspr2 (red) and Kv1.2 (green). **(A,A′)** Kv1.2 is detected at the AIS of a PV^+^ cell in the stratum pyramidale (Pyr). **(B–C)** Kv1.2 is localized at the AIS of SST^+^ neurons in the stratum oriens **(B,B′)** and stratum pyramidale **(C)**. AIS are indicated with arrows. Note that Kv1.2 is distributed more distally than ankyrinG along the axon **(B′)**. **(D,E)** Caspr2 and Kv1.2 are co-expressed in PV^+^ cells of the stratum pyramidale **(D)** and SST^+^ cells in the stratum oriens **(E)**. Single confocal section **(A–C)** and maximum intensity of 5-z steps of 1.31 μm (insets in panel **A′,B′**) or 4-z steps of 830 nm **(D,E)**. Scale bar: 15 μm.

Next, we analyzed the pattern of expression of Kv1.2 in the CA1 region of the hippocampus (Figure 10). We found that Kv1.2 was localized at the AIS of PV^+^ cells in the pyramidal layer (Figure 10A,A′) and SST^+^ cells in the stratum pyramidale (Figure 10C) and oriens (Figure 10B), but distally as compared to ankyrinG (Figure 10B′). We observed that PV^+^ cells in the pyramidal layer (Figure 10D) and SST^+^ cells located in the stratum oriens (Figure 10E) were positive for both Caspr2 and Kv1.2.

Our results show that the Kv1 complex proteins are enriched in the GABAergic neurons of the CA1 hippocampal region *in vivo*. Both PV and SST inhibitory neurons specifically express Kv1.2, TAG-1, and Caspr2, similarly to our *in vitro* findings. Moreover, a layer-specific expression of TAG-1 can be observed for SST and PV neurons that may indicate a specialization of these cells associated with myelination.

## Discussion

The Kv1 channels play a prominent role in repolarising the axon after action potential initiation at the AIS and in regulating propagation at the juxtaparanodes (Trimmer, 2015). During myelination, these channels are progressively enriched at the juxtaparanodes and at early stages, when the paranodal junctions have not yet formed or stabilized, Kv1 transiently localizes at nodes and paranodes (Vabnick et al., 1999; Hivert et al., 2016) and may be directly involved in speeding repolarisation to allow trains of action potentials. The dynamic distribution and precise sub-compartmental profile of Kv1 is thought to play an essential role in the developing axons switching from a continuous to a saltatory mode of conduction. In the present study, we show that in hippocampal cell cultures, the Kv1.2 subunits are selectively expressed all along GABAergic axons including the AIS before myelination, together with clusters of Nav1 channels and ankyrinG. Inhibitory axons are also highly enriched in molecules originally identified as part of the Kv1 complex at the juxtaparanodal regions of myelinated fibers, including the CAMs TAG-1, Caspr2, and ADAM22, as well as the scaffolding protein 4.1B. Cultures from TAG-1- or protein 4.1B-deficient mice indicate that the expression of TAG-1, Caspr2 and protein 4.1B is interdependent whereas the distal distribution of the Kv1.2 subunits is maintained in the absence of TAG-1 or protein 4.1B. *In vivo*, only subsets of SST and PV GABAergic neurons are positive for TAG-1 in the juvenile rat hippocampus, including the SST cells in the stratum oriens and the PV neurons in the stratum pyramidale, which also express Kv1.2 channels. This accurate distribution of ion channels and associated molecules along pre-myelinated axons may be crucial to regulate firing during development.

### Axonal Distribution of the Kv1 Complex in Inhibitory Hippocampal Neurons

In myelinated axons, the proper distribution of the Nav1 and Kv1 channels is strongly dependent on CAMs at nodal, paranodal, and juxtaparanodal regions. Specifically, a complex of TAG-1, Caspr2, and protein 4.1B mediates axo-glial contacts at juxtaparanodes and is required for Kv1 channel clustering (Poliak et al., 2003; Traka et al., 2003). However, TAG-1 and Caspr2 are dispensable for the trapping of the Kv1 channels at the AIS (Ogawa et al., 2008; Duflocq et al., 2011). Here, we observed that TAG-1 and ADAM22 are enriched at the AIS and expressed all along pre-myelinated inhibitory axons, whereas Caspr2 and protein 4.1B display a more distal distribution. Furthermore, taking advantage of KO mouse lines, we showed that Caspr2, TAG-1, and 4.1B are interdependent for their distribution along inhibitory axons. The expression of Caspr2 was strongly reduced along the inhibitory axons from TAG-1 or 4.1B KO hippocampal cultures. TAG-1 and protein 4.1B bind the ectodomain and the cytoplasmic tail of Caspr2, respectively, and may be required for its stabilization at the axonal membrane. We hypothesize that the association of Caspr2 with protein 4.1B may stabilize Caspr2 at the axolemma by inhibiting its internalization (Bel et al., 2009; Pinatel et al., 2017). Next, we observed that in contrast to Caspr2, the distal distribution of TAG-1 in inhibitory axons was reduced only in a small percentage of 4.1B-deficient neurons. We previously reported the level of TAG-1 is not altered in the brain of 4.1B KO mice (Cifuentes-Diaz et al., 2011). TAG-1 is trapped at the AIS independently of Caspr2 which is not enriched at that site. Our data suggest that TAG-1 may be associated with ADAM22 at the AIS and also along the axon. Indeed, we recently reported that TAG-1 can be associated with ADAM22 using co-immunoprecipitation experiments and that the two CAMs are sorted together in axonal transport vesicles (Hivert et al., 2019). However, the persistence of Kv1.2 subunits along the axons in TAG-1- and protein 4.1B-deficient cells reveals that the Caspr2/TAG-1/4.1B complex may be dispensable for the distal distribution of Kv1 channels. This indicates that the Kv1.2 tetramers which are axonally transported with their Kvß2 accessory subunits (Gu and Gu, 2010) are targeted to the axon independently from their associated CAMs. In the same way, the CAM neurofascin186, which is implicated in the initial nodal clustering of Nav1 channels in myelinated axons, is targeted at the axonal membrane through distinct mechanisms than the Nav1 channels (Zhang et al., 2012). In addition, neurofascin186 is not required for the clustering of pre-nodal complexes in pre-myelinated inhibitory neurons (Dr. Nathalie Sol-Foulon, personal communication). This early clustering of Nav1 channels linked with ankyrinG appears to be mediated by extracellular matrix proteins and soluble form of CAMs secreted by oligodendrocytes and it does not require the contact with the glial membrane. Our results indicate that this is unlikely to be the case for the clustering of the Kv1 channels at the nodes since we observed that the Kv1 channels are enriched at the AIS and thereafter uniformly distributed along the pre-myelinated axon, with the clustering of Kv1 being only detected where the myelin segments contact the axon. Interestingly, such a contact-dependent clustering of Kv1 channels has also been reported using co-cultures of hippocampal neurons with TAG-1 expressing HEK cells (Gu and Gu, 2011).

We further show that both the Kv1.1 and Kv1.2 subunits are expressed in GABAergic neurons whereas the Kv1.4 subunit is only localized at the AIS of excitatory neurons in hippocampal cell culture. However, Kv1.1 is faintly expressed along the inhibitory axons while it is strongly detected as intracellular vesicles. In this context, it is interesting to note that Kv1.4, which contains an ER export signal, has been shown to induce the cell surface targeting of Kv1.1 (Manganas and Trimmer, 2000; Lai and Jan, 2006; Jenkins et al., 2011). Therefore, these results suggest that Kv1 channels in inhibitory axons in hippocampal cell culture may mainly consist of Kv1.2 subunits. Importantly, the different composition of Kv1 tetramers allows distinct thresholds of channel activation (Bagchi et al., 2014).

### *In vivo* Selective Expression of TAG-1 in Subtypes of GABAergic Neurons in the Hippocampus

Recent reports have highlighted the possibility that different subtypes of GABAergic neurons could be myelinated. Long-range projecting inhibitory neurons connecting hippocampus with extra-hippocampal areas are known to be myelinated (Jinno et al., 2007; Melzer et al., 2012). This is the case of SST neurons in the stratum oriens of CA1, which project to the subiculum and to the entorhinal cortex. Surprisingly, it has been recently reported that a substantial fraction of myelin, both in mouse and human neocortex, belongs to GABAergic inhibitory neurons, in particular fast-spiking PV interneurons (Micheva et al., 2016; Stedehouder et al., 2017). The PV cells in the CA1 stratum pyramidale of hippocampus have been described to be frequently myelinated at 8–12 weeks of age, mainly on the proximal axonal segments, independently of their morphological subtypes (i.e., basket or bi-stratified) (Stedehouder et al., 2017). PV interneurons are high energy demanding cells, for which it is logical to think that myelin may provide axonal metabolic support (Kann et al., 2014; Krasnow and Attwell, 2016). Our results indicate that TAG-1 is exclusively expressed by some subtypes of GABAergic neurons in the rodent hippocampus, namely the PV cells of the pyramidal layer and the SST neurons of the stratum oriens in 3-week old rats. It remains to be precisely determined whether this selective expression of TAG-1 as a juxtaparanodal component may be associated with the myelinated fate of both local PV interneurons in the stratum pyramidale and long-range projecting SST neurons of the stratum oriens. Supporting this possibility, we observed that TAG-1 is enriched at the AIS of some SST^+^ cells of the stratum oriens, proximal to myelin segments.

The inhibitory axons of the PV and SST cells display pre-nodal clusters of Nav1 channels, which have been shown to promote acceleration of conduction in pre-myelinated axons as analyzed in cultured hippocampal neurons (Freeman et al., 2015). The presence of Nav1 clusters along pre-myelinated PV interneurons as observed *in vitro* and also *in vivo* (Freeman et al., 2015) may be related with the results of Hu and Jonas (2014), which showed a gradual increase of Na^+^ conductance in the distal axon of PV interneurons. The authors suggest that a high density of Na^+^ channels could be necessary for ensuring both speed of propagation and fast-spiking action potentials in unmyelinated axons. In addition, it has been shown that Kv1.1 channels localized at the AIS dampen excitability and prevent high frequency discharge at normal subthreshold levels in fast-spiking GABAergic cortical neurons (Goldberg et al., 2008). The Kv1.1 subunit is co-localized with ankyrinG at the AIS of PV basket cells in the hippocampus *in vivo* (Campanac et al., 2013). Here, we observed that the Kv1.2 subunit and TAG-1 are expressed at the AIS of both in PV and SST cells in the CA1 region of the hippocampus. Our data show the expression of a high density of Kv1 channels associated with CAMs occurring along pre-myelinated axons of subtypes of PV interneurons during development. With this respect, it will be important to analyze the physiological role of the high axonal content of the Kv1 complex in PV interneurons before myelination or in demyelinated pathological conditions.

## Data Availability

All datasets generated for this study are included in the manuscript and/or the Supplementary Files.

## Ethics Statement

This study was carried out in accordance with the recommendations of the European and Institutional guidelines for the care and use of laboratory animals and approved by the local authority (laboratory’s agreement number D13-055-8, Préfecture des Bouches du Rhône).

## Author Contributions

GB conceived and performed the experiments, analyzed the data, and wrote the manuscript. BH conceived and performed the experiments and analyzed the data. LG and DK provided the reagents and KO mice and discussed the data. VC discussed the data and provided the financial support. CF-S conceived and performed the experiments, analyzed the data, wrote the manuscript, provided the financial support, and supervised the study.

## Conflict of Interest Statement

The authors declare that the research was conducted in the absence of any commercial or financial relationships that could be construed as a potential conflict of interest.
