# Supplementary figures and images for: Selective Axonal Expression of the Kv1 Channel Complex in Pre-myelinated GABAergic Hippocampal Neurons
Source: Front Cell Neurosci. 2019 May 16;13:222. doi: 10.3389/fncel.2019.00222 (PMC6535494; doi:10.3389/fncel.2019.00222)

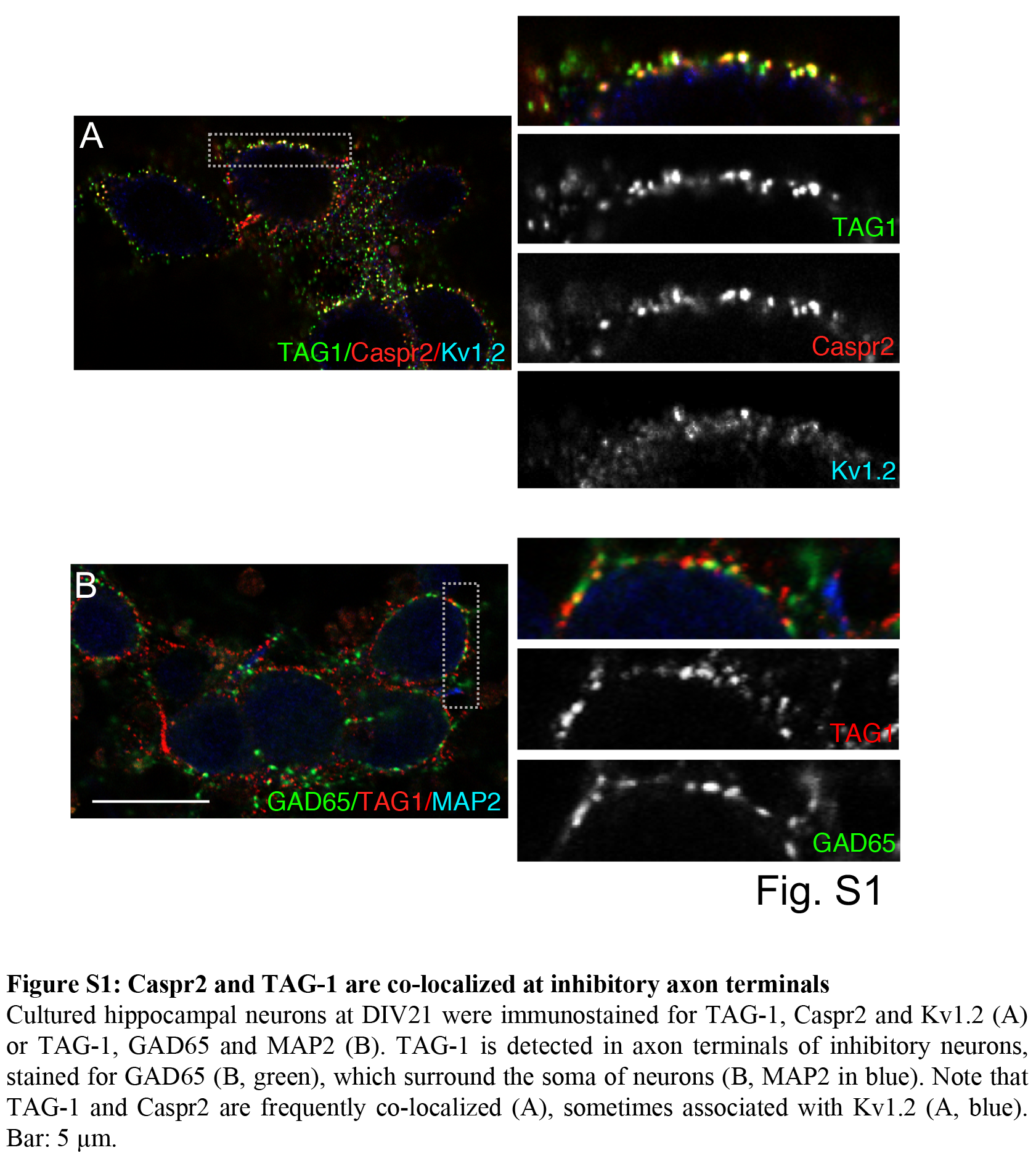

Supplement: Supplementary file 1 [file Image_1.TIF]
